# Stable and momentary psychosocial correlates of everyday smoking: An application of Temporal Self-Regulation Theory

**DOI:** 10.1007/s10865-021-00248-4

**Published:** 2021-08-06

**Authors:** Christopher M. Jones, Benjamin Schüz

**Affiliations:** Institute of Public Health and Nursing Research, University of Bremen, Grazer Str. 4, 28359 Bremen, Germany

**Keywords:** Self-regulation, Ecological Momentary Assessment, Smoking, Health behaviour, Temporal Self-Regulation Theory

## Abstract

Smoking is one of the leading causes of non-communicable disease mortality and morbidity. Smoking behaviour is determined by both stable, person-level (e.g., motivation, nicotine dependence) and variable, situation-level factors (e.g., urges, cues). However, most theoretical approaches to understanding health behaviours so far have not integrated these two spheres of influence. Temporal Self-Regulation Theory (TST) integrates these person-level and situation-level factors, but has not yet been comprehensively applied to predicting smoking behaviour. We use Ecological Momentary Assessment to examine the utility of TST in predicting daily smoking. 46 smokers reported individual and environmental cues right after smoking and at random time points during the day. Cognitions, self-control, past behaviour, and nicotine dependence were assessed at baseline. Multi-level logistic regressions show that smoking is largely guided by momentary cues, but individual motivation can buffer their influence. This suggests that TST is a useful integrative approach to understand modifiable determinants of smoking and thus intervention targets.

## Introduction

With one billion smokers around the world and some eight million deaths per year, smoking remains one of the most significant preventable causes of morbidity and early mortality (Reitsma et al., 2017). However, the effects of behavioural interventions to support smoking cessation remain small to medium in size at best (Lancaster & Stead, 2017; Mottillo et al., 2009). Therefore, it is important to advance our understanding of smoking as individual behaviour and identify it’s modifiable determinants.

Most theoretical approaches to understanding, predicting and modifying health-related behaviours such as smoking have focused on individual motivation as the proximal determinant of behaviour (e.g., intentions in the reasoned action approach RAA; Fishbein & Ajzen, 2011). These so-called social-cognitive approaches suggest that intentions are the most proximal predictor of behaviour: Here, intentions are considered the focal motivational component, i.e., a construct that concentrates potentially different sources of motivation towards the engagement in behaviour. Such different sources of motivation could, for example, be attitudes or social norms in the RAA. However, empirically, a “gap” remains between what people intend to do and what they actually do (McEachan et al., 2011, 2016; Sheeran & Webb, 2016). This gap has been hypothesized to reflect that social-cognitive approaches primarily account for motivation and do not specify the processes involved in translating motivation into action sufficiently—which could be one reason for the later addition of volitional constructs such as perceived behavioural control (Hagger et al., 2018; Rise et al., 2008).

It has been suggested that challenges in translating motivation into behaviour are related to moderating factors on the level of the person (e.g., trait-like self-regulatory capacity; Tangney et al., 2004) as well as momentary factors (e.g., cues to smoke, urges; Hall & Fong, 2013). Research on smoking behaviour has emphasized the importance of these potentially moderating factors. Daily and regular smoking is driven by clinical factors, most notably cravings/urges related to nicotine dependence (Berli et al., 2015; Ferguson & Shiffman, 2009; Sayette, 2016; Shiffman et al., 2007), but also by situation-varying determinants such as social and environmental factors (e.g., Serre et al., 2015; Shiffman et al., 2002; Thrul et al., 2014), momentary goals/intentions (e.g., Bolman et al., 2018; Vangeli et al., 2011), and affect (e.g., Leventhal, 2010; Shiffman et al., , 1996, 2008).

### Temporal Self-Regulation Theory

**Fig. 1:**
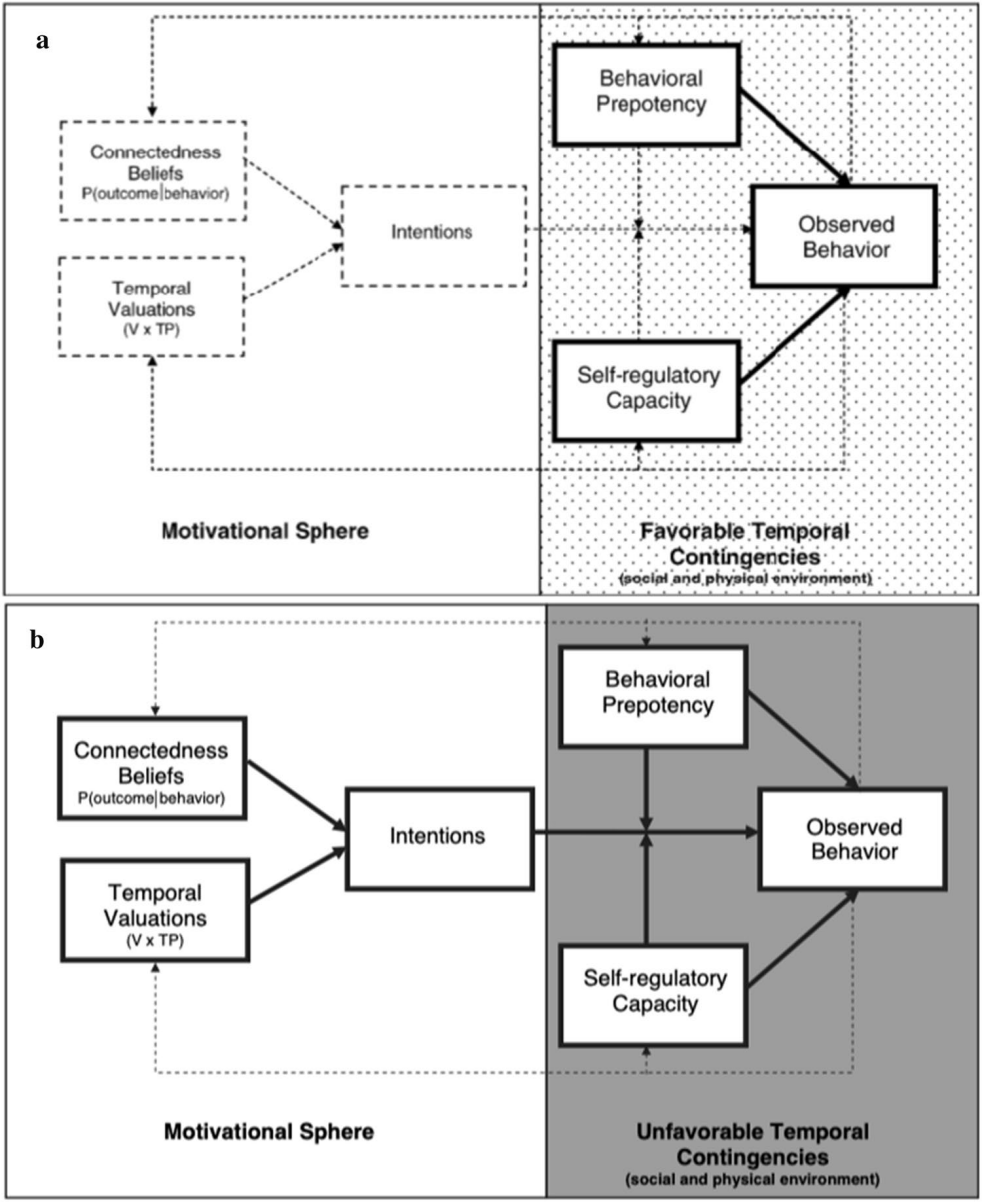
Conceptual diagram for repetitive behaviours in a supportive (**a**) and non-supportive ecological context (**b**), adapted from Hall and Fong (2007). Broken arrows denote weaker hypothesized effects

Temporal Self-Regulation Theory (TST) integrates person-level and situation-level factors and assumes that behavioural self-regulation happens in two parallel so-called spheres: A *motivational sphere* which describes the cognitive determinants of forming behavioural intentions, and a *momentary sphere* which describes the momentary contextual influences that impact in situ behavioural self-regulation (Fig. 1).

Within the *motivational sphere*, TST emphasizes the importance of subjective perceptions of the temporal distance of potential benefits and costs of any behaviour (Hall & Fong, 2007). It is assumed that immediate (i.e., smaller temporal distance) behaviour-consequence contingencies exert a stronger influence on behaviour than long-term contingencies. Accordingly, intentions result from beliefs about the outcome of a given behaviour (*connectedness beliefs*) and its valuation (*value beliefs*) as well as the perceived temporal distance to the outcome (*temporal valuations*). Intentions then serve as the most proximal motivational predictor of behaviour. However, intentions are theorized to result not only from the cognitions described above but also from momentary influences (e.g., current temptations, current social norms; Hall & Fong, 2015).

The *momentary sphere* describes the factors that influence behaviour directly or indirectly, here in particular by affecting the degree to which intentions can be translated into behaviour in a given moment. These factors include *behavioural prepotency*, which integrates past behaviour (e.g. behavioural frequency or intensity, habit strength, dependence intensity), potential biological predispositions, and cues within the momentary environment (e.g. social cues, availability) or the individual herself (e.g. craving/urges, affect). This construct accordingly allows the integration of physiological factors such as nicotine dependence within the wider behavioural framework of the theory. Additionally, cognitive resources enable individuals to regulate behaviour in line with intentions/goals in the given moment (*self-regulatory capacity*). This implies several potential interactions: While the translation of intentions into behaviour can be modified by behavioural pre-potency and self-regulatory capacity, these two factors can also interact (e.g., self-regulatory capacity allowing an individual to withstand current cue exposure). However, Hall and Fong (2007) propose that the importance of each factor might differ, depending on the nature of the behaviour (repetitive or discrete) and the ecological context (supportive or non-supportive; Fig. 1).

By specifying behavioural self-regulation as influenced by both motivational and momentary factors, TST not only offers the potential to improve the prediction of behaviour but also to identify pre- and post-intentional factors influencing successful behavioural self-regulation which could be targeted by interventions. So far, TST has been found useful in explaining a broader set of lifestyle behaviours (e.g., Booker & Mullan, 2013), the consumption of sugar-sweetened beverages (Moran & Mullan, 2020), or dietary behaviours (Elliston et al., 2017; Evans et al., 2017). However, only one of these studies (Elliston et al., 2017) has examined TST by assessing the momentary context, with real-time measurement using Ecological Momentary Assessment (EMA; Shiffman et al., 2008), whereas most other studies relied on summary self-reports of momentary influence factors. Furthermore, despite the potential of including relevant clinical person-level variables such as nicotine dependence in the prediction, no previous study has applied TST to predicting smoking behaviour.

## Aims of the present research

Therefore, the purposes of the present study were to examine the utility of Temporal Self-Regulation Theory in predicting smoking behaviour and to identify potentially modifiable determinants. Specifically, we aim to examine both spheres proposed by TST. Firstly, within the motivational sphere, we aim to examine whether the cognitions proposed within the TST are associated with intentions to quit (the focal ‘outcome’ of the motivational sphere). Secondly, we aim to examine whether momentary intentions, behavioral pre-potency and self-regulatory capacity are associated with smoking behaviour. We also include all relevant interactions described above.

Specifically, we examine the following research questions:

1. Motivational sphere: *Are smoking-related connectedness and value beliefs as well as the perceived temporal proximity of consequences associated with intentions to quit smoking at baseline?*
2. Momentary sphere: *Are momentary intentions, aspects of behavioural pre-potency (dependence related momentary urges, past behaviour, momentary cues), self-regulatory capacity, and their interactions associated with momentary smoking?*

The present study uses a hierarchical study design with multiple and frequent measurements within participants that matches the multilevel structure implied by TST (motivational and momentary sphere).

## Method

### Overview

The present study used Ecological Momentary Assessment applied to smoking (EMA; Ferguson & Shiffman, 2011) with smartphones (Motorola Moto-C; Android), running study-specific software. Participants logged every cigarette they smoked and reported individual and environmental cues right after smoking and at random (non-smoking) time points during the day for three weeks. The study protocol was approved by the University Ethics Committee (2018-05).

### Participants

Participants were recruited using a combination of social media (facebook and a local online job portal) as well as flyer and poster advertisements in a large city in northern Germany. Eligibility criteria included being 18 years or older, smoking cigarettes only and smoking at least 10 cigarettes per day. Participants received 10€ after the first lab visit and an additional 20€ after completing the study with a minimum compliance rate of 75%.

The final sample consisted of 46 daily smokers (mean age = 37.00, SD = 16.80, range: 18–74), of whom 53% were female. Participants had started smoking at an average age of 15.49 years (SD = 3.83), smoked 19.00 cigarettes a day on average (SD = 8.55), and 38.50% had partners who also smoked. More than one-third of the participants (40.82%) had graduated university, while 16.33% reported lowest secondary school qualifications. The initial sample had consisted of 53 participants but was reduced due to low levels of study participation, participants only responding to cigarette prompts or technical difficulties with the study smartphones.

### Design and procedure

This study employed a mixed EMA design using event-based and time-based assessments during the 21-day monitoring period. Before the start of the monitoring period, participants visited the lab to fill in baseline questionnaires (e.g. demographics, previous smoking behaviour, trait self-control) and receive hands-on training with the study smartphones and the software used for data collection. A second lab visit after three to four days was held to ensure compliance with the study protocol and verify no technical difficulties had occurred (troubleshooting). The final meeting was used for debriefing and participant reimbursement.

During the 21-day monitoring phase, participants were asked to log every cigarette, using the study smartphone. To reduce the burden on participants, only a pre-specified proportion of these logs (sampling probability 50%; Schüz et al., 2016; about 4–7 per day) were followed up by a set of further questions (event-based assessments). Additionally, participants were beeped at seven random time points during the day and presented the same set of questions (time-based assessment). To ensure sufficient spacing between prompts, random prompts were only allowed to be sent at least 30 min after a cigarette was logged. To ensure the random nature of the random prompts, participants were only allowed to answer within the next 20 min.

To amend the registered number of cigarettes smoked per day, participants were able to log any cigarette they had not reported before during brief morning and evening reports.

### Measures

#### Baseline assessment

We assessed all cognitions relevant to the motivational sphere at baseline. *Temporal contingency* was assessed following Hall and Fong (2007): Participants were asked to indicate when they expected costs and benefits of (a) smoking as well as (b) quitting to occur, from 1 = “when I think about smoking” to 10 = “several decades after smoking”. In line with recent research on TST (Evans et al., 2017), *value beliefs* were assessed by asking participants whether they rated quitting as positive or negative (“Do you agree with the following statement? ‘Quitting smoking would be good for me!’”; anchored: “not at all!”—“absolutely!”), and *connectedness beliefs* by asking how likely they perceived the expected outcome of quitting (“Do you agree with the following statement? ‘Quitting reduces the likelihood of serious illness!’”; anchored: “not at all!”—“absolutely!”). *Intention to quit* at baseline was assessed by asking how strongly they agreed with the statement “I intend to quit smoking.”. Participants answered all three questions on a 10-point visual analogue scale (anchored: “not at all!”—“absolutely!”).

We assessed *behavioural pre-potency* as a multifaceted combination of the frequency of past behaviour (average number of cigarettes per day) and the Fagerstrom Test for Nicotine Dependence (FTND; Heatherton et al., 1991) assessing current nicotine dependence. This is consistent with previous studies (e.g. Black et al., 2017) and recommendations by the authors of TST (Hall & Fong, 2007).

*Self-regulatory capacity* was assessed using the 13-item trait-measure, the Brief Self-Control Scale (Tangney et al., 2004). The full scale showed acceptable internal consistency (M = 2.94, SD = 0.53, α = 0.79﻿).

#### Momentary assessment (random and event-contingent prompts)

Intending to ensure comparability, we used the same item as at baseline to measure *momentary intention to quit* during the 21-day monitoring period (“I intend to quit smoking.”). The item was again presented as a 10-point visual analogue slider scale (anchored: “not at all!”—“absolutely) developed for convenient use on smartphone touch-displays.

*External and internal situational cues* were logged using the same items during time-based (random) as well as event-based (smoking) assessments. This resulted in a comparable set of cues and thus predictors. We asked participants to report five different situational cues: momentary levels of affective valence (one item; “how do you feel right now?”; answers ranging from “very bad!” to “very good!”, 5-point scale) and arousal (one item; “how aroused do you feel right now?”; answers ranging from “not at all!” to “very strongly!”, 5-point scale), current urge to smoke (one item, 5-point scale), and whether they could see other people smoking in their group or just in view (each separately coded as “no” = − 1, yes = “1”). These cues represent different facets of behavioural pre-potency (Hall & Fong, 2007) and have previously been associated with smoking (Ferguson & Shiffman, 2009; Kassel et al., 2003; Shiffman et al., 2002). Before analyzing the data, we subtracted 1 from all Likert- and visual analogue scale values to create a more meaningful range starting at zero agreement with each statement.

### Analyses

Firstly, we estimated a linear regression model predicting intentions to quit at *baseline*. Secondly, we estimated a multilevel linear regression model with random intercepts for each participant (for further details on data structure see below) predicting *momentary intentions* quit during EMA. We included value and connectedness beliefs as well as the participants’ evaluations of the timing of benefits and costs of smoking/quitting as predictors in *both models*. Additionally, we included participants’ age, gender, trait self-control, nicotine dependence strength and average smoking.

To examine everyday momentary smoking behaviour, we used a logistic regression approach. We regressed type of assessment (random/non-smoking, coded as “0”, or a smoking assessment, coded as “1”) on both stable person-level predictors as well as momentary predictors as proposed by TST. The model included fixed main effects for average smoking and the FTND sum score, trait self-control (all level-2), the number of the day of study participation, momentary intention to quit, momentary affective valence and arousal, momentary urge to smoke, and presence of other people currently smoking in view and in one’s group (all level-1). In line with TST, we also included interaction terms for momentary intention (level-1) with urge to smoke, environmental cues, affective valence and arousal (all level-1) and all level-2 predictors. We also included an interaction term for momentary urge (level-1) and trait self-control (level-2).

As each participant completes several assessments each day as well as during the whole study period, the data has a hierarchical structure with measurements nested within participants. To account for this non-independency, we adopted a multilevel approach with random intercepts, treating assessments (level-1 units) as nested within participants (level-2 units). We report the odds of smoking (compared to a random assessment) at any measurement point. To decompose within- and between-subject effects for level-1 predictors, we included a) each level-1 predictor’s person-mean and b) each level-1 predictor centered on this mean (Curran & Bauer, 2011). All level-2 predictors were grand-mean centered. We allow slopes to vary for all level-1 predictors (Barr et al., 2013).

All predictors used and their respective level of measurement can be found in Supplement 1. Within *lme4* (R package), we used the *bobyqa* optimizer and the default Laplace approximation.

To test whether any predictor assessed at baseline was associated with participants’ compliance and could thus lead to biased estimates, we computed each participant’s random prompt compliance and regressed it on participants’ intentions to quit, nicotine dependence strength, average cigarettes smoked per day, trait self-control, age, connectedness and value beliefs as well as temporal contingencies.

All analyses were conducted using R (Version 3.6.1; R Core Team, 2018) and the R-packages *lme4* (Version 1.1.19; Bates et al., 2015), *multilevel* (Version 2.6; Bliese, 2016), *psych* (Version 1.8.10; Revelle, 2018), *mitml* (Version 0.4-1; Grund et al., 2021).

### Multiple imputation

To provide sensitivity analyses, we created a new data frame matching the overall full-compliance number of random prompts and the expected number of cigarette prompts based on the within-subject ratio of both types of prompts. We then used a model-based imputation procedure in line with our multilevel regression model including all interaction terms and random effects to impute the missing data (Enders et al., 2019). This procedure resulted in 25 datasets with 14,259 observations each. Further information on the imputation model and procedure can be found in the imputation script (Supplement 4). Please note that all reported estimates based on this procedure are pooled across models.

## Results

### Assessments

Overall, participants logged a total of 7,571 cigarettes and completed 1,669 smoking as well as 1,712 random assessments. In total 3,381 full assessments were completed—49.36% of these assessments were smoking assessments.

### Compliance

We found no significant associations of intentions to quit, nicotine dependence strength, average cigarettes smoked per day, trait self-control, age, connectedness and value beliefs as well as expected temporal contingencies with random prompt-related compliance rates (see Supplement 3).

### Motivational sphere: associations with intention to quit

We had hypothesized that intentions to quit smoking (at baseline: M = 2.46, SD = 1.09) were associated with connectedness and value beliefs as well as the perceived temporal proximity of benefits and costs. Stronger value beliefs and more distant benefits of smoking were significantly associated with stronger intentions to quit smoking at baseline (Table 1), but not during momentary assessments (Table 2). None of the additional predictors were significantly associated with intentions to quit.

**Table 1:** Summary of linear regression model predicting baseline intention to quit: parameter estimates, confidence intervals and fit statistics

Predictor	*b*	*b* 95% CI (LL, UL)	*beta*	*beta* 95% CI (LL, UL)	Fit
Intercept	− 2.64	(− 7.57, 2.29)			
Connectedness beliefs	0.00	(− 0.17, 0.17)	0.00	(− 0.32, 0.32)	
**Value beliefs**	0.24*	(0.01, 0.46)	0.35	(0.02, 0.69)	
Distance quitting costs	0.11	(− 0.10, 0.32)	0.16	(− 0.15, 0.48)	
Distance quitting benefits	0.02	(− 0.12, 0.16)	0.05	(− 0.28, 0.37)	
Distance smoking costs	− 0.01	(− 0.15, 0.13)	− 0.03	(− 0.38, 0.32)	
**Distance smoking benefits**	0.26*	(0.03, 0.49)	0.38	(0.05, 0.72)	
Nicotine dependence	0.12	(− 0.05, 0.30)	0.24	(− 0.09, 0.58)	
Average smoking	− 0.02	(− 0.07, 0.03)	− 0.16	(− 0.53, 0.21)	
Trait self-control	0.19	(− 0.81, 1.18)	0.06	(− 0.24, 0.35)	
Age	0.01	(− 0.01, 0.03)	0.14	(− 0.23, 0.51)	
Gender	− 0.12	(− 0.71, 0.47)	− 0.06	(− 0.35, 0.23)	
					*R*^*2*^ = .305
					95% CI (.00, .35)

A significant *b*-weight indicates the beta-weight is also significant. *b* represents unstandardized regression weights. *beta* indicates the standardized regression weights. *LL* and *UL* indicate the lower and upper limits of a confidence interval, respectively

^*^p < 0.05, **p < 0.01, ***p < 0.001, bold: *p* < 0.05

**Table 2:** Summary of multilevel linear regression model predicting momentary intention to quit: parameter estimates and confidence intervals of each covariate, random effects

Predictor	Estimate		95% CI (LL, UL)
Intercept	3.47**	(.087, 6.08)	
Connectedness beliefs	0.08	(− 0.29, 0.45)	
Value beliefs	0.44	(− 0.06, 0.94)	
Distance quitting costs	− 0.14	(− 0.62, 0.34)	
Distance quitting benefits	− 0.16	(− 0.47, 0.15)	
Distance smoking costs	− 0.00	(− 0.32, 0.32)	
Distance smoking benefits	− 0.13	(− 0.64, 0.38)	
Nicotine dependence	− 0.47	(− 1.30, 0.35)	
Average smoking	− 0.12	(− 1.04, 0.80)	
Trait self-control	0.16	(− 0.56, 0.88)	
Age	− 0.00	(− 0.06, 0.05)	
Gender	0.80	(-0.53, 2.12)	
**Random effects:** σ^2^ = 1.17 τ_00_ _participant_ = 5.84 N_participant_ = 48			

*LL* and *UL* indicate the lower and upper limits of a confidence interval, respectively

^*^p < 0.05, **p < 0.01, ***p < 0.001, bold: *p* < 0.05

### Momentary sphere: associations with everyday smoking

**Fig. 2:**
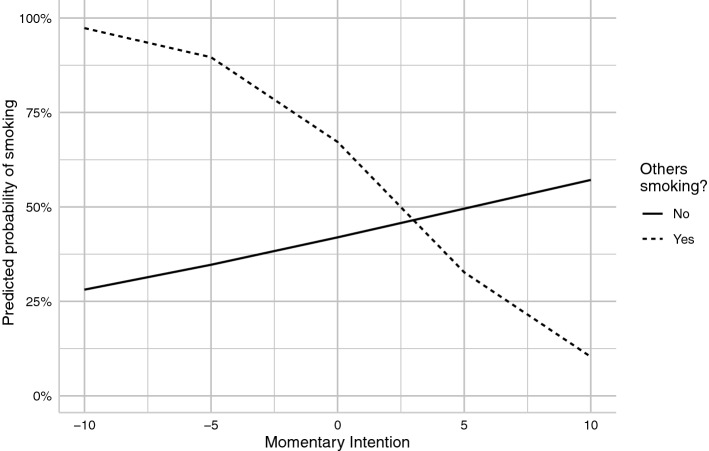
Model-based predicted probabilities of type of prompt as a function of whether others are smoking in view (levels: “no”, coded as − 1; “yes”, coded as 1) and momentary intentions to quit (Lüdecke, 2018). Shaded intervals depict the 95% confidence intervals around model predictions

None of the person-level predictors were associated with the odds of smoking in everyday life (Table 3). Of the momentary predictors (level-1), the presence of others smoking in participants’ view or in their group significantly increased the odds of smoking (at person-mean level of intentions). However, the effect of others smoking in view differed as a function of momentary intention to quit: In case of lower momentary intentions to quit, the presence of others smoking in view was associated with higher odds of smoking—this was not the case when intentions were higher (see Fig. 2; predicted probabilities).

**Table 3:** Summary of multilevel logistic regression model predicting type of assessment (random or smoking): Parameter estimates, standard errors, odds ratios with confidence intervals of each covariate

	Model 1: Listwise deletion of missing values		Model 2: imputation (M = 25)
	Estimate (SE)	Odds Ratio (95% CI)	Pooled Estimate (SE)
*Level—1 variables*			
Intercept	0.79 (1.02)	2.21 (0.30, 16.30)	0.682 (0.248)
Day in study	− 0.01 (0.01)	0.99 (0.98, 1.01)	− 0.000 (0.001)
Momentary intention (ws)	− 0.20 (0.10)	0.82 (0.67, 1.00)	− 0.017 (0.014)
Momentary urge (ws)	0.16 (0.10)	1.18 (0.96, 1.44)	0.001 (0.001)
Momentary affective arousal (ws)	0.10 (0.07)	1.11 (0.97, 1.26)	0.024 (0.015)
Momentary affective valence (ws)	− 0.03 (0.05)	0.97 (0.88, 1.08)	− 0.012 (0.022)
Others smoking (in group) ***	0.58*** (0.10)	**1.78 (1.46, 2.17)**	0.119*** (0.024)
Others smoking (in view) ***	0.52*** (0.10)	**1.69 (1.39, 2.05)**	0.084*** (0.025)
*Level—1 interactions*			
Mom. intention × Mom. urge	0.09* (0.04)	1.10 (1.01, 1.19)	0.000 (0.000)
Mom. intention × Smoking (group)	− 0.07 (0.07)	0.94 (0.82, 1.01)	0.002 (0.009)
**Mom. intention × Smoking (view)**	− 0.17* (0.08)	**0.84 (0.72, 0.99)**	− 0.025* (0.011)
Mom. intention × aff. valence	− 0.06 (0.04)	0.94 (0.87, 1.03)	− 0.003 (0.010)
Mom. intention × aff. arousal	− 0.05 (0.05)	0.95 (0.87, 1.05)	− 0.004 (0.008)
*Level—2 variables*			
Momentary intention (bs)	0.04 (0.04)	1.05 (0.96, 1.14)	0.001 (0.013)
Momentary urge (bs)	0.00 (0.01)	1.00 (0.99, 1.01)	− 0.000 (0.002)
Momentary aff. Arousal (bs)	− 0.30 (0.23)	0.74 (0.47, 1.18)	− 0.049 (0.056)
Momentary aff. Valence (bs)	0.08 (0.26)	1.09 (0.65, 1.81)	0.008 (0.070)
Average cigarettes per day (CPD)	0.03 (0.10)	1.03 (0.84, 1.26)	0.002 (0.003)
Nicotine dependence (FTND)	− 0.00 (0.10)	1.00 (0.82, 1.20)	− 0.017 (0.012)
Trait self-control	− 0.08 (0.14)	0.93 (0.71, 1.21)	− 0.092 (0.110)
*Cross-level interactions (L1 x L2)*			
Mom intention × Self-control	− 0.01 (0.04)	0.99 (0.92, 1.07)	− 0.029 (0.021)
Mom intention × CPD	0.04 (0.04)	1.04 (0.97, 1.12)	0.001 (0.001)
Mom intention × FTND	− 0.05 (0.05)	0.95 (0.87, 1.04)	− 0.004 (0.004)
Mom. urge × Self-control	0.00 (0.07)	1.00 (0.87, 1.15)	− 0.002 (0.002)
Smoking (group) × Self-control	− 0.11 (0.10)	0.90 (0.74, 1.08)	− 0.081 (0.080)
Smoking (view) × Self-control	− 0.05 (0.09)	0.95 (0.80, 1.12)	− 0.022 (0.067)

Parameter estimates on level-2 variables are interactions with the intercept

^*^p < 0.05, **p < 0.01, ***p < 0.001, bold: *p* < 0.05 for model 1 and model 2, ‘ws’ denotes within-subject centered and ‘bs’ between-subject centered momentary predictors

On a side note, participants’ mean momentary intentions to quit were significantly associated with baseline intentions to quit (*r*(46) = 0.38, *t* = 2.79, *p* = 0.007).

## Discussion

### Overview

This study was the first to examine the utility of Temporal Self-Regulation Theory (TST; Hall & Fong, 2007) in testing associations with everday smoking behaviour. Using Ecological Momentary Assessment (EMA; Shiffman et al., 2008) to tap into the motivational and momentary spheres influencing behaviour, we find that rather stable intentions only play a minor role in predicting everyday smoking. Instead, it is the momentary environmental and social cues that shape behaviour, with social cues exerting the strongest influence. However, our results also highlight the interactions between stable and momentary factors as the influence of environmental cues to smoke was buffered by stronger momentary intentions to quit.

Thus, we find preliminary evidence for the utility of TST to integrate different streams of research and explain momentary smoking behaviour. By incorporating cognitions, cognitive resources, physiological determinants (nicotine dependence), and ecological factors, TST offers an elaborate perspective on the person-environment interaction that shapes everyday smoking behaviour.

### Motivational sphere

In line with previous research (Hall et al., 2012), we find subjective temporal valuations and value beliefs significantly associated with intentions to quit *at baseline*. However, neither was significantly associated with momentary intentions to quit during EMA. This decreasing strength of associations could be attributed to an increasing temporal distance in measurement (Ajzen, 1996), especially taking into account that recent research has highlighted day-level variability in intentions to quit as well as repeated transitions from smoking as usual to trying to reduce smoking or even quitting (Hughes et al., 2005, 2014). In line with these findings, only 25% of the participants constantly reported no intention to quit during *momentary assessments* (see Supplement 1), with most other participants showing a considerable amount of variation in their motivation to quit (ICC = 0.84). Still, neither average intentions nor momentary within-subject fluctuations thereof—as the most proximal motivational predictor—exerted a significant influence on momentary smoking behaviour. Although in contrast with recent research on other health-related behaviours (e.g. Black et al., 2017; Moran & Mullan, 2020), these findings can be interpreted as in line with TST: For smoking as a highly repetitive behaviour within mostly supportive contexts (only 10.74% of prompts were reported to be logged under a smoking ban or others’ restrictions), a decreased influence of cognitions on behavioural self-regulation could be expected (Hall & Fong, 2007, Fig. 1).

### Momentary sphere

Within the *momentary sphere*, behavioural pre-potency shows a dominant influence on in situ self-regulation as environmental and social cues were significantly associated with smoking. While recent research on TST has discussed different ways to conceptualize behavioural pre-potency on a person-level—ranging from general measures of past behaviour frequency (Booker & Mullan, 2013) to habit strength (Evans et al., 2017), here, we also account for the clinically relevant effects of nicotine dependence strength (Ferguson & Shiffman, 2009). We thus expand on recent conceptualizations by combining person-level nicotine dependence (FTND) as well as momentary-level urges to smoke (Ferguson & Shiffman, 2009) with past behaviour strength (Hall & Fong, 2013) and the momentary assessment of environmental, social and additional internal cues—based on the assumption of “cue-contingent automaticity” (Orbell & Verplanken, 2010). Combining such more *general* with smoking-*specific* aspects of pre-potency provides additional insight into the specific effects. In line with these considerations, we find that environmental (OR = 1.69, 95% CI: 1.39, 2.05) and social cues (OR = 1.78, 95% CI: 1.46, 2.17) drive smoking behaviour in everyday life. Such cues can acquire incentive salience and shape a smoker’s attention, subsequent behaviour and thus increase the probability of behavioural enactment (Berridge & Robinson, 2016). With high levels of automaticity, little to no conscious awareness is needed (Wood et al., 2002). As participants had started smoking early and smoked 19.02 cigarettes per day (SD = 8.55), high levels of automaticity and thus cue-contingent behaviour could be assumed. On the other hand, seeing others smoke not only provides a momentary behavioural model but also renders the environment rather supportive of the behaviour. Taking a social cognitive perspective for example, one could assume that seeing others smoke, especially when in one’s own group, should result in changes to the momentary descriptive norms (e.g., Schüz et al., 2018). These changes should in turn result in altered momentary intentions. We tested whether the presence of others was related to momentary intentions but found no significant association.

In addition, we find support for the interaction of momentary intentions with behavioural pre-potency (social cues). This is in line with one of the key assumptions of TST, the *interconnectedness of its predictors within the momentary sphere*. Here, higher momentary intentions are able to partially buffer the effects of environmental cues (Fig. 2). Although TST currently only assumes either moderating effects of behavioural pre-potency on the intention-behaviour relationship or a generally reduced role of intentions regarding repetitive behaviours in rather supportive contexts, our findings might point towards a necessary adjustment of these assumptions: The buffering of the effects of momentary cues fits well with dual-process conceptions of self-regulation (Inzlicht et al., 2020). Along these lines of thought, momentary intentions to quit are a necessary requirement for the presence of conflict between both systems. Subsequently, stronger intentions could support a stronger deliberate and controlling influence of cognitive functioning (System II; Hofmann et al., 2009). This potential *integration of a dual-systems perspective on self-regulation* has already been highlighted by other recent research on TST (Black et al., 2017; Moran & Mullan, 2020) and might be crucial as we find no evidence for an effect of trait self-control on momentary smoking. However, it is important to note that we used a self-report measure of trait self-control instead of executive functioning tasks as an indicator of self-regulatory capacity, which could be considered a key limitation of the study (see below).

### Limitations

There are some methodological limitations to the current study. Firstly, to limit participant burden, we had to keep momentary assessments short. This means that we did not include a momentary measure of self-control or, as Hall and Fong (2013) suggest, a measure of momentary executive function. There is an ongoing debate on the use and interchangeability of self-report and behavioural measures with recent publications highlighting low correlations between both for self-regulation (Eisenberg et al., 2019; Saunders et al., 2018). Conceptually, behavioural measures have mostly been designed to maximize within-person variability, while also reducing between-person variability in order to produce robust experimental effects (Dang et al., 2020). Within our measurement scheme, we thus used a self-report measure to capture between-person differences of repeated/average and domain-unspecific performance (Wennerhold et al., 2020). However, past research on TST has also used self-report measures as well as cognitive tasks to measure self-regulatory capacity—with no associations with behavioural outcomes or other predictors (e.g. self-report measure: Evans et al., 2017; behavioural measure: Fulham & Mullan, 2011). Future research should take into account within-person variability of self-regulatory capacity. Including not only subjective individual (trait-) differences but within-person momentary fluctuations could help further distinguish the role of self-regulatory capacity within TST (e.g., direct effect or interacting with other predictors). Secondly, repeated momentary assessment and the resulting burden on participants restrict the use of more than a few items per construct and, accordingly, the use of most validated self-report measures. For example, we had to collapse positive and negative affect into one dichotomous item—both have been studied separately before. These feasibility requirements raise questions regarding reliability and validity. Thirdly, as the present study was correlational, we can only report associations between different factors but not causal mechanisms connecting them—associations might be due to other influences not assessed. Fourthly, the present study is well powered to detect effects of level-1 predictors (momentary assessment), but less so for level-2 predictors (person-level) as well as cross-level interactions. This could lead to biased standard errors and thus inferences (Maas & Hox, 2005). Fifthly, as no validated scales to assess value beliefs and connectedness beliefs exist, we created new items. However, these items might have induced some response bias due to their wording. Sixthly, rather unsurprisingly considering that participants were daily smokers, intentions to quit at baseline were very low. Although intentions to quit showed sufficient variability at baseline and during EMA, this should still be interpreted as a limitation. Future research should thus target samples of non-daily smokers to cover more of the range of observed smoking behaviour.

### Implications

The present study offers new insights into the application of TST and preliminary evidence for its utility in explaining momentary smoking behaviour.

From a theoretical standpoint, three implications arise: Firstly, TST offers added value to the understanding of health behaviour self-regulation by embedding and integrating motivational factors and cognitive resources into the momentary environment. Secondly, higher temporal resolution of measurement in the present study adds precision to conceptual relationships within TST. Thus, future modifications of TST should include more potential interactions between different predictors as already indicated by recent updates (Hall & Fong, 2013). This could, thirdly, result in TST being consistent with several other theories of self-regulation (i.e. dual-process models, resource models, trait models of impulse control).

From a practical standpoint, improved understanding of *how* both spheres proposed by TST interact and which factors contribute most to everyday smoking has implications for intervention development. With the influence of environmental and social cues, interventions could aim to strategically alter environments. For example, introducing more restricted smoking areas to public spaces and also making smoking areas less central and thus visible and more effortful to reach, might result in less environmental/social cues and also altered descriptive norms. While those interventions operate on a policy level, changes could also be achieved by training smokers and successful quitters to avoid specific places with high rates of smokers present—just as proposed by recent concepts of effortless self-control (Adriaanse et al., 2014). As strong intentions to quit showed potential to buffer the effects of environmental cues, evaluated techniques to increase intention strength should be adopted. This emphasizes the potential of complex interventions using both spheres of influence and their connections between and within.

TST is still an emerging theory and a “work in progress”. Therefore, additional research and evidence are needed to draw stronger conclusions about the different determinants’ influence on smoking behaviour. However, our results emphasize the role of the momentary environment (central to behavioural pre-potency) as a primary intervention target and point to the need for complex behavioural interventions as well as a policy based on a thorough theoretical understanding of what guides smoking behaviour.

## Data Availability

All data and analysis scripts that support the findings of this study will be made available upon publication at https://osf.io/rns7g/.

## References

[CR1] Adriaanse MA, Kroese FM, Gillebaart M, De Ridder DTD (2014). Effortless inhibition: Habit mediates the relation between self-control and unhealthy snack consumption. Frontiers in Psychology.

[CR2] Ajzen I, Gollwitzer PM, Bargh JA (1996). The directive influence of attitudes on behavior. The psychology of action: Linking cognition and motivation to behavior.

[CR3] Barr DJ, Levy R, Scheepers C, Tily HJ (2013). Random effects structure for confirmatory hypothesis testing: Keep it maximal. Journal of Memory and Language.

[CR4] Bates D, Mächler M, Bolker B, Walker S (2015). Fitting linear mixed-effects models using lme4. Journal of Statistical Software.

[CR5] Berli C, Ochsner S, Stadler G, Knoll N, Hornung R, Scholz U (2015). Volitional processes and daily smoking: Examining inter- and intraindividual associations around a quit attempt. Journal of Behavioral Medicine.

[CR6] Berridge KC, Robinson TE (2016). Liking, wanting, and the incentive-sensitization theory of addiction. American Psychologist.

[CR7] Black N, Mullan B, Sharpe L (2017). Predicting heavy episodic drinking using an extended temporal self-regulation theory. Addictive Behaviors.

[CR8] Bliese, P. (2016). Multilevel: Multilevel functions. Retrieved December 8, 2018, from https://CRAN.R-project.org/package=multilevel

[CR9] Bolman C, Verboon P, Thewissen V, Boonen V, Soons K, Jacobs N (2018). Predicting smoking lapses in the first week of quitting: An ecological momentary assessment study. Journal of Addiction Medicine.

[CR59] Booker L, Mullan B (2013). Using the temporal self-regulation theory to examine the influence of environmental cues on maintaining a healthy lifestyle. British Journal of Health Psychology.

[CR11] Curran PJ, Bauer DJ (2011). The disaggregation of within-person and between-person effects in longitudinal models of change. Annual Review of Psychology.

[CR12] Dang J, King KM, Inzlicht M (2020). Why are self-report and behavioral measures weakly correlated?. Trends in Cognitive Sciences.

[CR13] Eisenberg IW, Bissett PG, Zeynep Enkavi A, Li J, MacKinnon DP, Marsch LA, Poldrack RA (2019). Uncovering the structure of self-regulation through data-driven ontology discovery. Nature Communications.

[CR14] Elliston KG, Ferguson SG, Schüz B (2017). Personal and situational predictors of everyday snacking: An application of temporal self-regulation theory. British Journal of Health Psychology.

[CR15] Enders CK, Du H, Keller BT (2019). A model-based imputation procedure for multilevel regression models with random coefficients, interaction effects, and other nonlinear terms. Psychological Methods.

[CR16] Evans R, Norman P, Webb TL (2017). Using Temporal Self-Regulation Theory to understand healthy and unhealthy eating intentions and behaviour. Appetite.

[CR17] Ferguson SG, Shiffman S (2009). The relevance and treatment of cue-induced cravings in tobacco dependence. Journal of Substance Abuse Treatment.

[CR18] Ferguson SG, Shiffman S (2011). Using the methods of ecological momentary assessment in substance dependence research—Smoking cessation as a case study. Substance Use & Misuse.

[CR19] Fishbein M, Ajzen I (2011). Predicting and changing behavior: The reasoned action approach.

[CR100] Fulham E, Mullan B (2011). Hygienic food handling behaviors: Attempting to bridge the intention-behavior gap using aspects from temporal self-regulation theory. Journal of Food Protection.

[CR20] Grund, S., Robitzsch, A., & Luedtke, O. (2021). mitml: Tools for multiple imputation in multilevel modeling. R package version 0.4-1. Retrieved 11 November, 2020 from https://CRAN.R-project.org/package=mitml

[CR21] Hagger MS, Polet J, Lintunen T (2018). The reasoned action approach applied to health behavior: Role of past behavior and tests of some key moderators using meta-analytic structural equation modeling. Social Science & Medicine.

[CR22] Hall PA, Fong GT (2007). Temporal self-regulation theory: A model for individual health behavior. Health Psychology Review.

[CR23] Hall PA, Fong GT, Hall P (2013). Temporal self-regulation theory: Integrating biological, psychological, and ecological determinants of health behavior performance. Social neuroscience and public health.

[CR24] Hall PA, Fong GT, Yong H-H, Sansone G, Borland R, Siahpush M (2012). Do time perspective and sensation-seeking predict quitting activity among smokers? Findings from the International Tobacco Control (ITC) Four Country Survey. Addictive Behaviors.

[CR25] Hall PA, Fong GT (2015). Temporal self-regulation theory: A neurobiologically informed model for physical activity behavior. Frontiers in Human Neuroscience.

[CR26] Heatherton TF, Kozlowski LT, Frecker RC, Fagerstrom K-O (1991). The Fagerström test for nicotine dependence: A revision of the Fagerstrom Tolerance Questionnaire. British Journal of Addiction.

[CR27] Hofmann W, Friese M, Strack F (2009). Impulse and self-control from a dual-systems perspective. Perspectives on Psychological Science.

[CR60] Hughes JR, Keely JP, Fagerstrom KO, Callas PW (2005). Intentions to quit smoking change over short periods of time. Addictive Behaviors.

[CR28] Hughes JR, Solomon LJ, Naud S, Fingar JR, Helzer JE, Callas PW (2014). Natural history of attempts to stop smoking. Nicotine & Tobacco Research.

[CR29] Inzlicht, M., Werner, K. M., Briskin, J. L., & Roberts, B. (2020). *Integrating Models of Self-Regulation* [Preprint]. PsyArXiv. 10.31234/osf.io/dpjye 10.1146/annurev-psych-061020-105721 33017559

[CR30] Kassel JD, Stroud LR, Paronis CA (2003). Smoking, stress, and negative affect: Correlation, causation, and context across stages of smoking. Psychological Bulletin.

[CR31] Lancaster T, Stead LF (2017). Individual behavioural counselling for smoking cessation. Cochrane Database of Systematic Reviews.

[CR32] Leventhal AM (2010). Do individual differences in reinforcement smoking moderate the relationship between affect and urge to smoke?. Behavioral Medicine.

[CR33] Lüdecke D (2018). ggeffects: Tidy Data Frames of Marginal Effects from Regression Models. Journal of Open Source Software.

[CR34] Maas CJM, Hox JJ (2005). Sufficient sample sizes for multilevel modeling. Methodology.

[CR35] McEachan RRC, Conner M, Taylor NJ, Lawton RJ (2011). Prospective prediction of health-related behaviours with the Theory of Planned Behaviour: A meta-analysis. Health Psychology Review.

[CR36] McEachan R, Taylor N, Harrison R, Lawton R, Gardner P, Conner M (2016). Meta-analysis of the reasoned action approach (RAA) to understanding health behaviors. Annals of Behavioral Medicine.

[CR37] Moran A, Mullan B (2020). Exploring temporal self-regulation theory to predict sugar-sweetened beverage consumption. Psychology & Health.

[CR38] Mottillo S, Filion KB, Bélisle P, Joseph L, Gervais A, O’Loughlin J, Paradis G, Pihl R, Pilote L, Rinfret S, Tremblay M, Eisenberg MJ (2009). Behavioural interventions for smoking cessation: A meta-analysis of randomized controlled trials. European Heart Journal.

[CR39] Orbell S, Verplanken B (2010). The automatic component of habit in health behavior: Habit as cue-contingent automaticity. Health Psychology.

[CR40] R Core Team. (2018). *R: A language and environment for statistical computing*. Vienna, Austria: R Foundation for Statistical Computing. Retrieved December 8, 2018, from https://www.R-project.org/

[CR41] Reitsma MB, Fullman N, Ng M, Salama JS, Abajobir A, Abate KH, Abbafati C, Abera SF, Abraham B, Abyu GY, Adebiyi AO, Al-Aly Z, Aleman AV, Ali R, Al Alkerwi A, Allebeck P, Al-Raddadi RM, Amare AT, Amberbir A, Gakidou E (2017). Smoking prevalence and attributable disease burden in 195 countries and territories, 1990–2015: A systematic analysis from the Global Burden of Disease Study 2015. The Lancet.

[CR42] Revelle, W. (2018). *Psych: Procedures for psychological, psychometric, and personality research*. Evanston, Il: Northwestern University. Retrieved December 8, 2018, from https://CRAN.R-project.org/package=psych

[CR43] Rise J, Kovac V, Kraft P, Moan IS (2008). Predicting the intention to quit smoking and quitting behaviour: Extending the theory of planned behaviour. British Journal of Health Psychology.

[CR44] Saunders B, Milyavskaya M, Etz A, Randles D, Inzlicht M (2018). Reported self-control is not meaningfully associated with inhibition-related executive function: A Bayesian analysis. Collabra: Psychology.

[CR45] Sayette MA (2016). The role of craving in substance use disorders: Theoretical and methodological issues. Annual Review of Clinical Psychology.

[CR46] Schüz B, Papadakis T, Ferguson SG (2018). Situation-specific social norms as mediators of social influence on snacking. Health Psychology.

[CR47] Schüz N, Eid M, Schüz B, Ferguson SG (2016). Immediate effects of plain packaging health warnings on quitting intention and potential mediators: Results from two ecological momentary assessment studies. Psychology of Addictive Behaviors.

[CR48] Serre F, Fatseas M, Swendsen J, Auriacombe M (2015). Ecological momentary assessment in the investigation of craving and substance use in daily life: A systematic review. Drug and Alcohol Dependence.

[CR49] Sheeran P, Webb TL (2016). The intention-behavior gap: The intention-behavior gap. Social and Personality Psychology Compass.

[CR50] Shiffman S, Paty JA, Gnys M, Kassel JA, Hickcox M (1996). First lapses to smoking: Within-subjects analysis of real-time reports. Journal of Consulting and Clinical Psychology.

[CR51] Shiffman S, Balabanis MH, Gwaltney CJ, Paty JA, Gnys M, Kassel JD, Hickcox M, Paton SM (2007). Prediction of lapse from associations between smoking and situational antecedents assessed by ecological momentary assessment. Drug and Alcohol Dependence.

[CR52] Shiffman S, Gwaltney CJ, Balabanis MH, Liu KS, Paty JA, Kassel JD, Hickcox M, Gnys M (2002). Immediate antecedents of cigarette smoking: An analysis from ecological momentary assessment. Journal of Abnormal Psychology.

[CR53] Shiffman S, Stone AA, Hufford MR (2008). Ecological momentary assessment. Annual Review of Clinical Psychology.

[CR54] Tangney JP, Baumeister RF, Boone AL (2004). High Self-control predicts good adjustment, less pathology, better grades, and interpersonal success. Journal of Personality.

[CR55] Thrul J, Bühler A, Ferguson SG (2014). Situational and mood factors associated with smoking in young adult light and heavy smokers. Drug and Alcohol Review.

[CR56] Vangeli E, Stapleton J, Smit ES, Borland R, West R (2011). Predictors of attempts to stop smoking and their success in adult general population samples: A systematic review. Addiction.

[CR57] Wennerhold L, Friese M, Vazire S (2020). Why self-report measures of self-control and inhibition tasks do not substantially correlate. Collabra: Psychology.

[CR58] Wood W, Quinn JM, Kashy DA (2002). Habits in everyday life: Thought, emotion, and action. Journal of Personality and Social Psychology.

